# Potentials and Challenges in Development of Vesicular Phospholipid Gel as a Novel Dermal Vehicle for Thymol

**DOI:** 10.3390/pharmaceutics17070854

**Published:** 2025-06-29

**Authors:** Sabina Keser, Zora Rukavina, Marica Jozić, Lea Pavlović-Mitrović, Magda Vodolšak, Kristina Kranjčec, Darija Stupin Polančec, Gordana Maravić-Vlahoviček, Jasmina Lovrić, Maja Šegvić Klarić, Željka Vanić

**Affiliations:** 1 Department of Pharmaceutical Technology, Faculty of Pharmacy and Biochemistry, University of Zagreb, A. Kovačića 1, 10000 Zagreb, Croatia or sabina.keser@pliva.com (S.K.); zora.rukavina@pharma.unizg.hr (Z.R.); jozicmarica0@gmail.com (M.J.); lpavlovicmitrovic@student.pharma.hr (L.P.-M.); mvodolsak@student.pharma.hr (M.V.); kkranjcec@student.pharma.hr (K.K.); jasmina.lovric@pharma.unizg.hr (J.L.); 2 R&D, PLIVA Croatia Ltd., TEVA Group, Prilaz Baruna Filipovića 25, 10000 Zagreb, Croatia; 3 Healthcare Facility Pharmacy Slavonski Brod, Vukovarska 10, 35000 Slavonski Brod, Croatia; 4 Department of Microbiology, Faculty of Pharmacy and Biochemistry, University of Zagreb, A. Kovačića 1, 10000 Zagreb, Croatia; dstupinpolancec@pharma.unizg.hr (D.S.P.); maja.segvic@pharma.unizg.hr (M.Š.K.); 5 Department of Biochemistry and Molecular Biology, Faculty of Pharmacy and Biochemistry, University of Zagreb, A. Kovačića 1, 10000 Zagreb, Croatia; gordana.maravic@pharma.unizg.hr

**Keywords:** vesicular phospholipid gel, liposomes, thymol, skin, dermal drug delivery, biocompatibility

## Abstract

**Background/Objectives:** Thymol, one of the main compounds of thyme essential oil, has shown promising effects in treating various skin disorders owing to its anti-inflammatory, antimicrobial and antioxidative activities. Due to its limited solubility in water, thymol is commonly used in higher concentrations to achieve a suitable therapeutic effect, which can consequently lead to skin irritation. To overcome these limitations, we incorporated thymol into a vesicular phospholipid gel (VPG), a novel semisolid dermal vehicle consisting of highly concentrated dispersion of phospholipid vesicles (liposomes). **Methods:** Thymol was successfully loaded into two VPGs differing in bilayer fluidity, which were characterized for the physicochemical and rheological properties, storage stability, in vitro release, ex vivo skin permeability, in vitro compatibility with epidermal cells, wound healing potential, and antibacterial activity against skin-relevant bacterial strains. **Results:** High pressure homogenization method enabled preparation of VPG-liposomes of neutral surface charge in the size range 140–150 nm with polydispersity indexes below 0.5. Both types of VPGs exhibited viscoelastic solid-like structures appropriate for skin administration and ensured skin localization of thymol. Although both types of VPGs enabled prolonged release of thymol, the presence of cholesterol in the VPG increased the rigidity of the corresponding liposomes and further slowed down thymol release. **Conclusions:** Loading of thymol into VPGs significantly reduced its cytotoxicity toward human keratinocytes in vitro even at very high concentrations, compared to free thymol. Moreover, it facilitated in vitro wound healing activity, proving its potential as a vehicle for herbal-based medicines. However, the antibacterial activity of thymol against *Staphylococcus aureus* and methicillin-resistant *S. aureus* was hindered by VPGs, which represents a challenge in their development.

## 1. Introduction

Research and use of herbal medicines have significantly grown during recent decades due to increased interest in pharmacological efficacy of medicinal plants and their favorable patient acceptability. Thymol, a monoterpenoid phenol and one of the main compounds of thyme essential oil, has shown potential for the treatment of various skin disorders because of its multiple activities, comprising antibacterial, antifungal, antiparasitic, antioxidative, anticancer, and anti-inflammatory effects [1,2,3,4]. However, thymol also increases skin permeability and porosity, acting as a penetration enhancer [5], which can consequently lead to difficulty in its localization within the skin. Thymol is available as colorless crystals with a specific odor that are easily soluble in ethanol, but very slightly soluble in water [6]. Its volatility, hydrophobic nature (log P 3.3) [7], and low biological stability are considered limitations for its wider use. To overcome these drawbacks and enable development of thymol formulation for dermal delivery, different strategies have been utilized.

Among numerous nanoformulations investigated for improving thymol’s bioavailability, like polymeric nanoparticles [8], polymeric micelles [9], liposomes [10], nanorods [11], and nanostructured lipid carriers [12], liposomes (phospholipid vesicles) are especially appealing due to their similarity with skin structures and physiological acceptability. In addition, various active ingredients, differing in size and lipophilicity (hydrophilic, hydrophobic, amphiphilic), can be entrapped or encapsulated in liposomes and released in a sustained and controlled manner. By optimizing their composition and preparation method, liposomes of suitable physicochemical properties (size, lamellarity, bilayer fluidity, surface charge) can be obtained facilitating either increased or reduced permeability of active substance into/through the skin [13,14]. Since liposomes are liquid dispersions, their administration to the skin can lead to rapid leakage of the nanoformulation from the site of application, limiting the effectiveness of the encapsulated active substance. Incorporation of liposomes into semisolid vehicles like hydrogel is a common approach for improving their retention at the topical site of administration, further prolonging the release of the encapsulated drug [15,16]. The ratio of liposomes embedded into a hydrogel is typically ranging from 10% *w*/*w* [17,18] up to 30% *w*/*w* [15,16], subsequently resulting in dilution of liposomally-encapsulated substance. This can be a problem when there is a need for delivery of higher doses of active ingredients, such as herbal medicines. Another obstacle for liposomal delivery of plant-based medicines is liposomes’ preparation methods. Namely, almost all methods are restricted by the concentration of phospholipids in liposome formulations (<40 mg/mL), limiting encapsulation of larger doses of active ingredients. These disadvantages can be defeated by vesicular phospholipid gel (VPG), an extremely concentrated phospholipid dispersion in water, exhibiting semisolid consistency. Freeze fracture electron microscopy studies revealed that VPG comprises tightly packed small unilamellar liposomes [19] and incredibly low portion of outer water phase. Therefore, full drug loading is obtained in comparison to liquid liposomal nanoformulations. In the presence of water, VPG easily transforms into liquid liposomal dispersions still enabling superior drug encapsulation [20]. Hence, VPGs can be used as semisolid depot formulation, but also as a storage form for liposomes [21].

VPGs were originally developed as depot nanoformulations for parenteral drug delivery [22,23,24]. Fang et al. [25] suggested its application for ophthalmic delivery of flurbiprofen in the treatment of uveitis. Very recently, Keser et al. [26] developed ciprofloxacin hydrochloride-loaded VPGs for the treatment of chronic skin infections.

Currently, VPGs have not yet been examined for delivery of plant-based medicines. Therefore, in this research, for the first time, the potential of VPG as a vehicle for dermal delivery of thymol was examined. The semisolid texture of VPG is suitable for dermal administration, allowing prolonged contact on the skin surface as a precondition for extended and controlled delivery of thymol. Liposomal encapsulation of thymol could reduce its penetration through the skin, allowing a localized effect of thymol, while phospholipids from VPG can be incorporated into disturbed epithelial layers, assisting the reepithelization (healing) effect. To explore these assumptions, two different thymol-loaded VPGs (T-VPGs), varying in bilayer fluidity, were prepared and evaluated for the physicochemical and rheological features, ex vivo skin permeability and in vitro release in conditions simulating skin administration. Biocompatibility of VPGs and wound healing potential were explored on in vitro keratinocytes model, while antimicrobial activity was tested against *Staphylococcus aureus* and clinical isolate of methicillin-resistant *S. aureus* (MRSA).

## 2. Materials and Methods

### 2.1. Materials

Lipoid S100, i.e., a soybean lecithin containing greater than or equal to 94% phosphatidylcholine (SPC), was obtained from Lipoid GmbH (Ludwigshafen, Germany). Thymol, cholesterol, 2,3,5-triphyenyltetrazolium chloride (TTC), 3-(4,5-dimethyltialzol-2-yl)-2,5-diphenyltetrazolium bromide (MTT), Sephadex G-50, ethylenediamine tetra acetic acid (EDTA), gentamicin, and dimethyl sulfoxide (DMSO) were purchased from Sigma-Aldrich (Darmstadt, Germany). Methanol, ethanol, and acetonitrile were procured from BDH Prolabo (Lutterworth, UK) and were of HPLC grade. Propylene glycol was from T.T.T. (Sveta Nedelja, Croatia). Müller–Hinton broth (MHB), tryptic soy broth (TSB) and tryptic soy agar (TSA) were obtained from Merck (Darmstadt, Germany). Fetal bovine serum (FBS) was provided by Biosera (Cholet, France), while Dulbecco’s Modified Eagle’s Medium (DMEM) was from Gibco Invitrogen (Waltham, MA, USA). Penicillin, streptomycin and amphotericin B mixture and phosphate-buffered saline (PBS, pH 7.4) without calcium and magnesium were procured by Lonza (Basel, Switzerland). Trypsin (0.125%) was purchased from Capricorn Scientific (Ebsdorfergrund, Germany).

Phosphate buffer solution, pH 5.0, used in in vitro release studies, was prepared by dissolving 2.72 g of potassium dihydrogen phosphate in 800 mL of water, adjusting the pH using 1 M potassium hydroxide solution, and adding water up to 1000 mL [27].

Phosphate-buffered saline (PBS), pH 7.4, used in ex vivo permeation studies, was composed of 0.19 g potassium dihydrogen phosphate, 2.98 g disodium hydrogen phosphate dihydrate, and 8.0 g sodium chloride in water, up to 1000 mL [27].

Water used in all experiments was distilled water.

### 2.2. Preparation of T-VPGs

Thymol was loaded into two VPGs, differing in (phospho)lipid composition, using the high-pressure homogenization method [26]. The procedure was as follows: SPC, with or without cholesterol, was added to a beaker containing a solution of thymol in propylene glycol (Table 1). The mixture was dispersed in water during continuous magnetic stirring (500 rpm, 1.5 h) at room temperature or at 50 °C (preparation with cholesterol). The hydrated mixtures of (phospho)lipids and thymol were then homogenized in a Microfluidizer LM20 (Microfluidics, Westwood, MA, USA) at 500 bars in one cycle to form T-VPGs. Thymol-free VPGs (empty VPGs) were prepared under the same conditions, but without thymol, and were used as controls in in vitro antimicrobial studies.

T-VPGs were subjected to pH determination using a Mettler Toledo pH meter (Greifensee, Switzerland) and electrode for semisolid formulations (Greifensee, Switzerland). Prior to the measurements, the electrode was calibrated using standard pH solutions (pH 4.0, pH 7.0, and pH 9.0) [16]. The measurements were performed in triplicate for each VPG.

The prepared VPGs were stored at 4 °C until their use. They were thermostated at room temperature (30–60 min) before evaluation.

Prior to physicochemical assessment of VPGs (size, surface charge, membrane elasticity, liposomes’ encapsulation efficiency) and estimation of their in vitro cytotoxicity, antibacterial activity, and in vitro wound healing effect, VPGs were converted to VPG-liposomes by gradually dispersing each VPG (1 g) in water up to 10 mL using a magnetic stirrer (600 rpm, 30 min). Conversion of VPGs containing cholesterol to liquid liposomal dispersions was performed at 50 °C.

### 2.3. Size, Zeta Potential, and Membrane Elasticity Determinations

Average diameters, polydispersity indexes and zeta potentials of VPG-liposomes were assessed on a Zetasizer Ultra (Malvern Panalytical, Malvern, UK). Photon correlation spectroscopy was employed for size determination. The samples of VPG-liposomes were diluted in water (1:20, *v*/*v*) to obtain optically semi-transparent samples suitable for size evaluation. The measurements were performed at a scattering angle of 90 ° and temperature set to 25 °C [27]. Electrophoretic light scattering was applied for zeta potential measurements using a disposable folded capillary cell [26].

Membrane elasticity was employed on a home-made device [28,29]. In brief, T-VPG liposomes were subjected to extrusion through 100 nm (r_p_) pore size polycarbonate membranes (Avestin, Mannheim, Germany) under constant pressure (2.5 bar) for 5 min. The mass of extruded liposomes in grams (J) was weighed and their mean diameters (r_v_) were determined by dynamic light scattering. The degree of VPG-liposomes’ membrane elasticity (E) was calculated using the equation:

E = J·(r_v_/r_p_)^2^ (1)

All measurements were performed over three independent experiments.

### 2.4. Encapsulation Efficiency Assessment

Encapsulation of thymol in T-VPG liposomes was assessed after separation of non-encapsulated from the liposomally-encapsulated thymol by minicolumn centrifugation method, according to the previously described procedure [29]. Briefly, T-VPG liposomes (0.4 mL) were applied to the top of Sephadex G-50 minicolumns, which were then centrifuged for 3 min at 2000 rpm (Eppendorf Centrifuge 5702 R, Wien, Austria) to isolate liposomally encapsulated thymol at the bottom of the centrifuge tubes. Subsequently, 0.5 mL of water (supplemented with 10% ethanol) was added to each minicolumn, which was again centrifuged (2000 rpm, 3 min), and the eluates were collected for analysis. The last procedure, including rinsing the minicolumns with water containing 10% ethanol, was repeated until all unencapsulated thymol was removed.

Quantification of liposomally-encapsulated and non-encapsulated thymol was performed on a Cary 60 UV/Vis spectrophotometer (Agilent Technologies, Santa Clara, CA, USA) at 274 nm. To create standard calibration curves, solutions of thymol in methanol or in the buffer, pH 5.0 containing 10% ethanol were used, both in the range of 5–100 μg/mL thymol (R^2^ = 0.9928, methanol; R^2^ = 0.9996, buffer, pH 5.0 with 10% ethanol).

The amount of encapsulated thymol was assessed in eluates containing only liposomally encapsulated thymol after dissolving phospholipids by addition of methanol. Fractions containing free thymol were determined in buffer, pH 5.0 containing 10% ethanol. Encapsulation of thymol and thymol recovery were calculated according to following equations:

(2) $$Encapsulation\;efficiency\left(\%\right)=\frac{LT}{LT+FT}\times 100,$$

(3) $$Thymol\;recovery\left(\%\right)=\frac{LT+FT}{totalT}\times 100$$

where LT is liposomal thymol, while FT is free (non-encapsulated) thymol. Total T represented both liposomally encapsulated and non-encapsulated thymol determined in an aliquot of the initial liposomal dispersion after the addition of methanol to solubilize (phospho)lipids.

The studies were conducted over three independent experiments.

### 2.5. In Vitro Release Studies

Dialysis tubing method was applied to monitor the release of thymol from T-VPGs [29]. The studies were performed under sink conditions at 32 °C using the phosphate buffer, pH 5.0 supplemented with 10% ethanol as release medium.

Sample of T-VPG, corresponding to 3 mg thymol, was placed in a dialysis bag (Medicell Membranes Ltd., London, UK; Mw cut-off 3500 Da) and dialyzed against 100 mL of release medium during constant stirring (300 rpm). At certain time intervals (1, 2, 3, 4, 5, 6, 24, 27, and 30 h), samples of dialysis medium (3 mL) were removed and immediately replaced with the fresh medium. The content of the thymol released was quantified spectrophotometrically (Section 2.4).

Free thymol dissolved in the mixture of ethanol and water (4/6, *v*/*v*) represented control and was tested in the same concentration as in the T-VPGs.

The studies were carried out over three independent experiments.

#### Mathematical Modelling of Thymol Release

The in vitro release data also served to estimate different kinetic models: Zero- and First-order, Higuchi, and Korsmeyer–Peppas models [30,31]:

Zero-order: Q = Q_0_ + k·t, (4)

First-order: Q/Q_0_ = 1 − e^−k·t^, (5)

Higuchi model: Q = k·t^1/2^, (6)

Korsmeyer–Peppas: Q/Q_0_ = k·t^n^. (7)

where Q denotes thymol mass released at time t, Q_0_ is the initial mass of thymol, k is the release constant of each model, and n is the release exponent indicating the release mechanism. When n is 0.43, Fickian mechanism is dominant, while for n values in the range 0.43–0.85, the release denotes non-Fickian mechanism [30,32].

### 2.6. Rheometry of T-VPGs

#### 2.6.1. Viscosity

Viscosity profiles of T-VPGs were determined on a Modular Compact Rheometer MCR 102 (Anton Paar GmbH, Graz, Austria) utilizing RheoCompass^TM^ Light software version 1.30. The system was fitted with a cone-plate measuring system (CP25, 25 mm diameter) and temperature plate P-PTD200 (Anton Paar GmbH, Graz, Austria). Gap was set at 0.102 mm, and samples were equilibrated at 25 and 32 °C. Rotational tests were performed in the shear rate range from 0.01 to 1000 s^−1^, at 30 points. Before each measurement began, the sample was left on the measuring system for 10 min to ensure that handling the sample did not affect the results.

#### 2.6.2. Amplitude Sweep Test

Oscillatory amplitude sweep tests were carried out a Modular Compact Rheometer MCR 102 (Anton Paar GmbH, Graz, Austria) equipped with RheoCompass^TM^ analysis software. A parallel-plate measuring system (PP25, 25 mm diameter) and temperature plate P-PTD200 were used, with gap set at 1 mm. Prior to the measurements, samples were stabilized at 32 °C for 10 min to ensure that handling did not cause any variation in the measurements. The amplitude range was set from 0.01 to 100%, with an angular frequency of 10 s^−1^.

The tests were performed over three independent experiments.

### 2.7. Ex Vivo Permeation Studies

Penetration/deposition of thymol from the different T-VPGs was tested on a full-thickness pig ear skin using Phenix RDS automated Franz-cells diffusion testing system (Teledyne Hanson, Chatsworth, CA, USA). Ears were acquired from the slaughterhouse immediately after animals were slaughtered. The hairs were carefully removed by shaving, and the skin was separated from the underlying tissue using a scalpel. Skin samples were rinsed with 0.9% NaCl solution (saline), gently dried by a soft paper and frozen at -20 °C until use. Prior to the testing, the skin was defrosted at room temperature and pre-equilibrated in the saline (10–15 min). Samples of the skin (1.0 mm thick) were sandwiched between donor and acceptor compartments of the Franz-cells (1.77 cm^2^ surface area), with epidermis oriented toward donor compartment. The acceptor compartment was filled with 15 mL of phosphate buffer (pH 7.4) containing 30% ethanol to ensure the solubility of thymol in the acceptor medium. The acceptor medium was magnetically stirred at 250 rpm, and the entire system was thermostated at 32 °C. Sample of T-VPG or control (free thymol), each equivalent to 0.4 mg thymol, was placed on the skin surface in donor compartment and closed. At certain time intervals (2, 10, 16, 24, and 36 h) the acceptor medium was removed (0.5 mL) and immediately replaced with the fresh medium. After 36 h, the skin surface was rinsed with methanol to determine the thymol accumulated on the skin surface. The amount of thymol retained within the skin was determined after extraction of shredded pieces of the skin with methanol [26]. Quantification of thymol deposited on the skin surface, within the skin and penetrated thorough the skin into acceptor medium was determined by HPLC. The studies were performed over three independent experiments.

#### HPLC Assay for Thymol

Determination of thymol in ex vivo permeation studies was performed by HPLC using 1260 Infinity II LC System (Agilent, Santa Clara, CA, USA). Kinetex^®^ C18 column (4.6 mm × 250 mm, particle size 5 μm, Phenomenex, Torrance, CA, USA) equipped with Security Guard column protection (Phenomenex, Torrance, CA, USA) was utilized for thymol separation. Mixture of acetonitrile and water (50/50, *v*/*v*) represented the mobile phase. The flow rate was 1.3 mL/min, and the oven temperature was maintained at 40 °C. Thymol was detected at 274 nm at a retention time of 4.4 min, while the total run time was 7 min. Methanolic solutions of thymol (2.5–50 μg/mL) were used to construct a standard curve (R^2^ = 0.9998). All samples for analysis were filtered through 0.22 μm polyethersulfone filters (Nantong FilterBio Membrane Co., Ltd, Nantong, China). The analysis was conducted in triplicate.

### 2.8. In Vitro Antibacterial Testing

Antibacterial activity of T-VPG liposomes was tested against *S. aureus* ATCC 6539 and clinical isolate of *S. aureus* MRSA MFBF 10679, obtained from the microbial collection of the Department of Microbiology, University of Zagreb Faculty of Pharmacy and Biochemistry. Their antimicrobial effect was examined in relation to free thymol (dissolved in a mixture of ethanol and water, 4/6, *v*/*v*) and gentamicin (inoculum control). Cultures of *S. aureus* in MHB were used as positive controls (growth controls), while negative control represented cultivation medium without bacteria. Empty (drug-free) VPGs and the solvent (ethanol/water, 4/6, *v*/*v*) were tested under the same conditions to determine possible antibacterial activity caused by VPGs ingredients or the solvent itself.

To determine the minimal inhibitory concentrations (MICs) of the T-VPG liposomes and free thymol, a two-fold microdilution assay was performed on a 96-well plate using MHB according to the National Committee for Clinical Laboratory Standards (NCCLS) standard protocol [33]. The testing was performed according to procedure reported by Rukavina et al. [28]. Briefly, cultures of *S. aureus* in broth were treated with T-VPG liposomes or free thymol in concentrations of thymol ranging from 16 to 2048 µg/mL, while gentamicin (control) was tested at concentrations 0.08–10 µg/mL. Following 24 h of incubation at 37 °C under aerobic conditions, the MICs were determined by applying 1% TTC reagent dissolved in sterile water [34]. The studies were carried out over two independent experiments and four technical replicates.

### 2.9. In Vitro Cytotoxicity Studies

In vitro cytotoxicity studies were performed to test biocompatibility of T-VPGs with human keratinocytes. For that purpose, T-VPGs were converted to liposomes (Section 2.2.) and non-liposomal thymol was separated by minicolumn centrifugation method. T-VPG liposomes prepared in this way were tested on monolayers of HaCaT cells (Cell Line Services, Eppelheim, Germany). For comparison, a solution of thymol in ethanol and water (3:7, *v*/*v*) was also tested.

#### 2.9.1. Cell Culturing

HaCaT cells were cultured in DMEM supplemented with 10% FBS and a mixture of penicillin, streptomycin and amphotericin B at 37 °C and 5% CO_2_, according to the manufacturer’s protocol. To determine possible cytotoxic effects of T-VPG liposomes the cells were seeded onto 96-well plates at a density of 10^4^ cells/well and allowed to reach confluence in 48 h [28].

#### 2.9.2. Treatment of the Cells and MTT Assay

T-VPG liposomes and thymol solution were dispersed in non-supplemented DMEM to attain thymol concentrations ranging from 7.83 to 4000 μg/mL. Prior to the treatment, the medium was removed, and the cells were washed with PBS (pH 7.4). T-VPG liposomes or thymol solution were added onto the cells at the corresponding concentrations and incubated for the following 24 h. As a negative control, the cells treated only with DMEM medium were used. MTT colorimetric assay was applied to assess in vitro cytotoxicity of the examined samples by determining metabolic activity of the treated keratinocytes [28]. In brief, 24 h after treatment with T-VPG liposomes and thymol solution, the treatment agents were removed, and the cells were washed with PBS, pH 7.4, followed by addition of supplemented DMEM and incubation for the next 24 h. Afterwards, MTT solution in DMEM (10 µL) was added to each well and incubated for 30 min at 37 °C. After removal of the medium, acidified isopropanol (100 µL) was applied to each well, and the amount of formazan was determined spectrophotometrically at 570 nm (Victor, PerkinElmer, Waltham, MA, USA). The metabolic activity of the keratinocytes was evaluated relative to that of the control group treated with DMEM.

The studies were carried out over three independent experiments and three technical replicates.

### 2.10. In Vitro Scratch Assay

The scratch test was employed to estimate the healing ability of T-VPGs in vitro. It was performed on human keratinocytes monolayer using the recently reported procedure [26]. The HaCaT cells were seeded onto 24-well plates (10^4^ cells/well) to reach confluence during 24 h of incubation in DMEM supplemented with 10% FBS and the mixture of mixture of antimicrobials (37 °C, 5% CO_2_). A horizontal line was drawn on the bottom of each well to ensure that an equal portion of the well with the scratch would be monitored. After 24 h the culture medium was removed and the fresh non–supplemented DMEM was added and incubated for the following 24 h. Afterwards, a straight scratch, simulating a wound, was made with a 200 μL pipette tip. The keratinocytes monolayer was washed with PBS (pH 7.4) to eliminate separated cells. The scratches were treated with T-VPG liposomes or thymol solution at the concentration of thymol being 500 μg/mL. As a control, non-treated cells incubated in the non–supplemented DMEM, were used. Closing the scratch (wound’) was measured over 24 h using an inverted microscope (5× magnification; Olympus CKX4, Olympus Europa Gmbh, Hamburg, Germany) equipped with a camera (Samsung, 16 MP, f/1.9, Seoul, Republic of Korea). The wound healing rate was determined based on the difference between the initial scratch and the surface of the wound after 24 h, using ImageJ software version 1.x (National Institutes of Health, Bethesda, MD, USA). The wound closure rate (WCR) was calculated using the equation:

WCR (%) = (A_0_ − A_24_)/A_0_ × 100, (8)

where A_0_ represented the initial scratch area, while A_24_ indicated the scratch area after 24 h [35].

The studies were performed over three independent experiments and three technical replicates.

### 2.11. Storage Stability Studies

T-VPGs were stored at 4 °C for 5 months and during this period mean diameters, polydispersity indexes and zeta potentials of the corresponding T-VPG liposomes were examined, as described in Section 2.3. The measurements were performed over three independent experiments.

### 2.12. Statistical Analysis

Statistical data analysis was performed using one-way ANOVA and Tukey’s multiple comparison test (comparison of three or more groups). When two groups were compared, the *t*-test was applied. Calculations were carried out with the GraphPad Prism 8.4.3. (GraphPad Software, Inc., San Diego, CA, USA). *p* < 0.05 was set as the minimum level of significance.

## 3. Results and Discussion

### 3.1. Characteristics of T-VPG Liposomes

**Figure 1:**
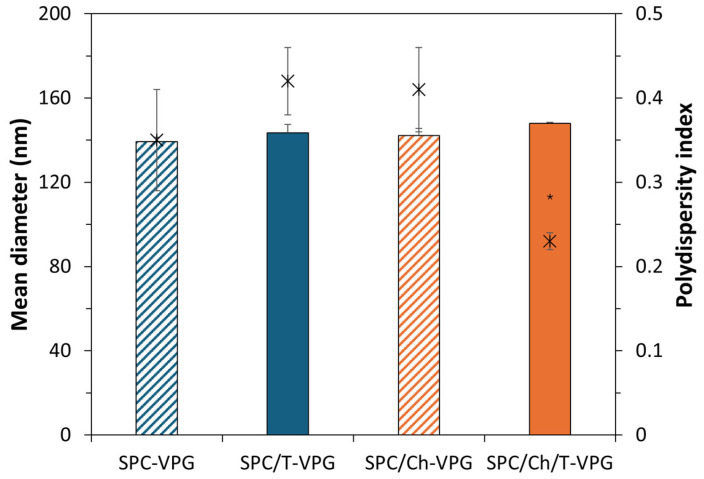
Mean diameters (columns) and polydispersity indexes (x symbol) of different VPG-liposomes, with or without thymol. The results denote mean ± S.D. (n = 3). * Statistically significant compared to SPC/T-VPG liposomes (ANOVA, *p* < 0.05).

Physicochemical properties of drug nanoformulations play a key role in the effectiveness of drug delivery by determining tissue penetration, drug release, interactions with the biological environment, and stability during storage [36,37]. These features are influenced by the composition of nanoformulation as well as the preparation method. Accordingly, one of the first goals during the development of T-VPGs was to examine the effect of (phospho)lipid composition and applied high-pressure homogenization on the physicochemical characteristics of VPG-liposomes, the structural units of VPGs (Figure 1).

Regardless of the presence of thymol or cholesterol, all VPG-liposomes were in the range of 140 to 150 nm, with polydispersity indexes between 0.23 and 0.42 (Figure 1). These results are expected given the homogenization of hydrated mixtures was carried out under high pressure (500 bar). They are consistent with the previous study in which VPG-liposomes containing hydrophilic drug ciprofloxacin hydrocholoride were evaluated [26]. In that investigation, VPG-liposomes of the same phospholipid composition had average diameters 130–145 nm, with the liposomes embedding cholesterol being negligibly larger, like in the present study (Figure 1).

**Figure 2:**
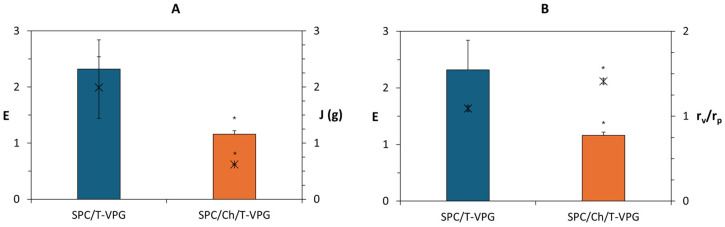
Bilayer’s elasticity of T-VPG liposomes. E, degree of bilayer elasticity (columns); J, mass (g) of extruded liposomes (x symbol); r_v_, mean diameters of extruded liposomes (nm); r_p_, membrane pore size (100 nm); r_v_/r_p_ (x symbol). * Statistically significant compared to SPC/T-VPG liposomes (*t*-test, *p* < 0.05).

Bilayer fluidity is another important physicochemical feature, which has been shown to significantly affect skin penetration, drug release pattern as well as stability of liposomes. As expected, the presence of cholesterol significantly enhanced the rigidity of VPG-liposomes (Figure 2) by its integration within the hydrophobic region of SPC bilayers. Thus, the calculated degree of bilayer elasticity (E) was twice as low as that of SPC/T-VPG liposomes. As in previous studies, E was commonly determined by the mass of extruded liposomes (J) through a 100 nm pore-sized membrane under constant pressure (Figure 2A) rather than by their size following extrusion (Figure 2B) [28,29,38].

**Figure 3:**
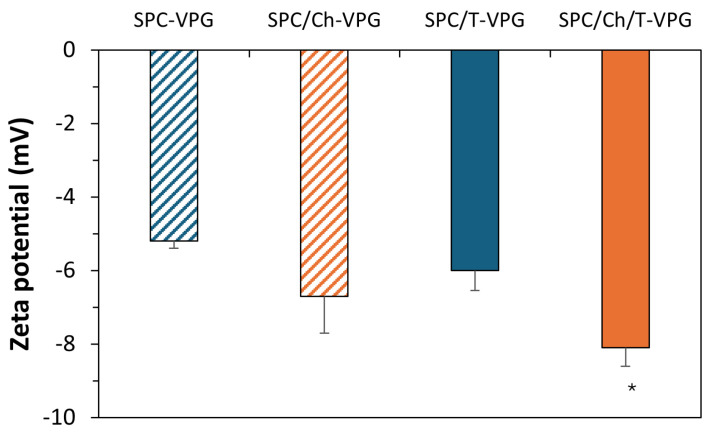
Zeta potentials of different VPG-liposomes, with or without thymol. The results denote mean ± S.D. (n = 3). * Statistically significant compared to SPC/T-VPG liposomes (ANOVA, *p* < 0.05).

All VPG-liposomes were slightly negatively surface-charged (almost neutral), with zeta potentials ranging from −5 to −8 mV (Figure 3). These results are determined by the characteristics of phosphatidylcholine (SPC), the main ingredient of VPGs, and are generally favorable for the localization of the encapsulated thymol in the skin. Namely, it has been confirmed that surface charge, in addition to bilayer fluidity, contributes to the penetration of liposomes into the skin. While cationic liposomes have been shown to exhibit the most pronounced skin penetration potential, followed by anionic liposomes with flexible membranes, neutral liposomes have demonstrated the highest ability to accumulate within the uppermost layers of the skin [39].

To ensure effective dermal delivery, thymol should be localized in a sufficient amount at the site of action. A crucial advantage of VPGs over classical liposomal dispersions lies in the fact that all the drug (active ingredient) is loaded within VPGs [20]. Because scratch test, in vitro cytotoxicity, and antibacterial testing impose the use of liquid formulations, T-VPGs were converted to the corresponding T-VPG liposomes. Non-liposomal thymol was separated (Section 2.4) to simulate conditions of T-VPG application where all thymol is loaded, and the amount of liposomally-encapsulated thymol was determined.

**Figure 4:**
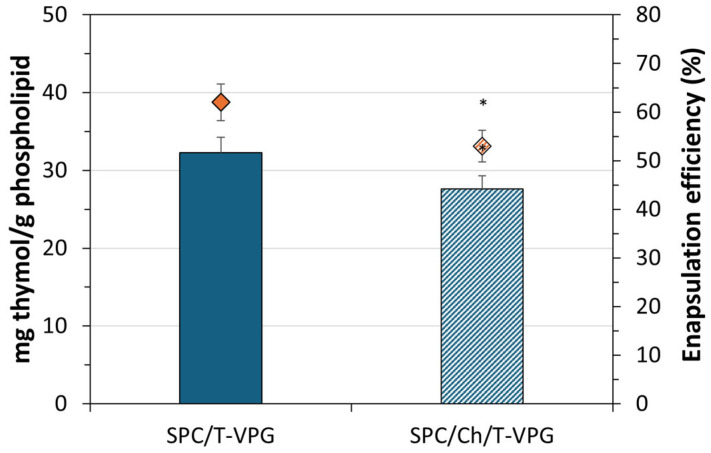
Encapsulation of thymol in VPG-liposomes, expressed as encapsulation efficiency (EE, rhombus sign) and mg of encapsulated thymol per g of (phospho)lipids used (columns). * Statistically significant compared to SPC/T-VPG liposomes (*t*-test, *p* < 0.05).

As shown in Figure 4, both T-VPG liposomes allowed satisfactory encapsulation of thymol, which was significantly higher than the encapsulation of a hydrophilic drug in VPG-liposomes [26]. The calculated thymol recoveries were in the range of 93 to 104%. Better encapsulation of thymol was achieved in SPC/T-VPG liposomes (62%, 32 mg/g phospholipid). This is probably a result of the greater concentration of SPC in SPC/T-VPG liposomes than in the nanoformulation containing cholesterol (Table 1). Furthermore, as thymol is a hydrophobic molecule, it probably competes with cholesterol within the lipid regions of SPC/Ch/T-VPG liposomes, resulting in lower encapsulation efficiency (53%, 28 mg/g (phospho)lipid (*p* < 0.05).

Several studies report almost complete thymol loading in liposomes (98–99%); however, due to the specific experimental setup used for determining encapsulation efficiency, which is not appropriate for a hydrophobic active ingredient, it is possible that the apparent encapsulation of thymol in those liposomes was overestimated [10,40]. On the other hand, Engel et al. [41] reported a small amount of encapsulated thymol, while Miranda-Cadena et al. [42] demonstrated similar encapsulation of thymol in liposomes as in this research.

### 3.2. Characteristics of T-VPGs 

**Figure 5:**
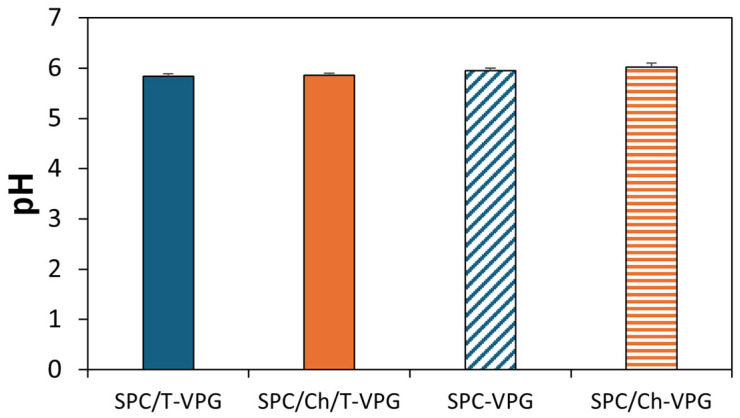
pH values of different VPGs. Values denote mean ± S.D (n = 3).

Physiological and anatomical properties of skin should be considered when designing a dermal drug nanoformulation. The applied nanoformulation should not disrupt the barrier function and physiology of skin. For instance, the pH of the healthy skin surface is between pH 4.1 and 5.8, depending on the part of the body [43]. It elevates in the elderly to pH 5.5–6.0 and significantly increases in infections to neutral or even alkaline pH [44,45]. Therefore, the use of formulations with a slightly acidic pH (4–6) is preferred for skin care and dermal therapy [46]. However, the pH of the nanoformulation should not adversely affect its stability or stability of the incorporated active ingredient. Since the constitutive elements of VPGs and liposomes have been shown to be most stable at pH 6–7 [47], one of the first goals during the development of T-VPGs was to achieve an appropriate pH that is consistent with the pH of healthy skin and conductive to liposome stability. Hence, the pH values of both prepared T-VPGs were 5.8 (Figure 5), which is suitable for skin application, while maintaining stability of liposomes.

Once T-VPG is applied dermally, it needs to be evenly distributed over the skin surface, where it should remain for a sufficient period to enable the release of the incorporated thymol as a prerequisite for its therapeutic effect. Such characteristics are closely related to the ingredients of the VPGs and their concentration. Therefore, rheological assessment and in vitro release studies are crucial for evaluating the properties of the prepared T-VPGs. Moreover, rheological studies assist in controlling the manufacturing processes of drug formulations, detecting changes that may occur during storage or transport, and evaluating the formulation’s behavior during administration [48,49]. Topical drug formulations may exhibit time- and temperature-dependent changes in viscosity that can impact the release profile of the active ingredient from the semisolid matrix and influence important formulation properties such as stability, appearance, spreadability, and retention at the application site [50]. Hence, the viscosity curves of T-VPGs were determined at 25 and 32 °C, corresponding to storage and skin surface temperatures, respectively.

**Figure 6:**
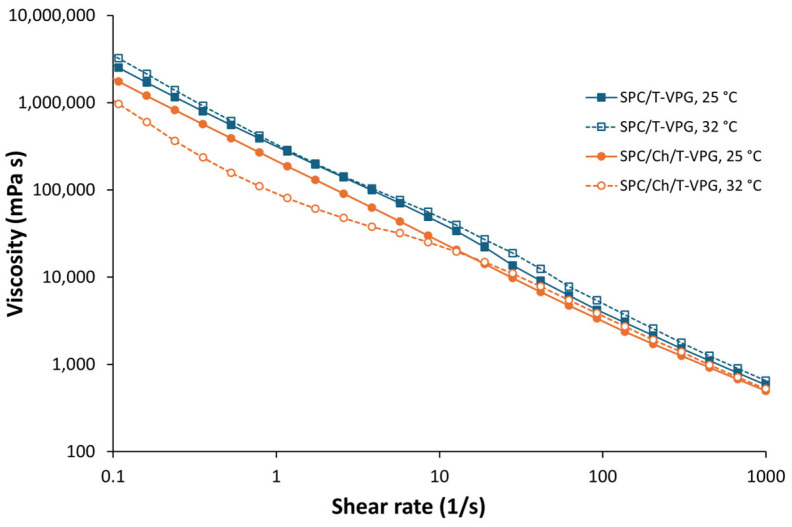
Viscosity profiles of T-VPGs at 25 and 32 °C.

Both T-VPGs exhibited non-Newtonian behavior, typical for pseudoplastic systems (Figure 6). The viscosity of the prepared T-VPGs depended on the shear rate. Regardless of the temperature at which the sample was measured, increasing the shear rate led to a decrease in viscosity, because of the disruption of the matrix structure caused by external force.

The viscosity curves of T-VPGs showed negligible differences, indicating comparable viscosity. Interestingly, SPC/Ch/T-VPG demonstrated a decrease in viscosity at 32 °C within the shear rate range of 0.1–10 s^−1^, probably due to the higher measurement temperature, as it is well-established that viscosity decreases with increasing temperature [51]. However, at higher shear rates, the influence of temperature was less noticeable (Figure 6). The slightly lower viscosity of SPC/Ch/T-VPG was probably caused by the lower SPC content compared to SPC/T-VPG (Table 1). This is in agreement with a recent study [26], where a VPG containing cholesterol was less viscous than the VPG composed of SPC alone.

To examine the effect of T-VPGs’ composition on the viscoelastic structure of the VPGs, oscillatory amplitude tests were performed. Amplitude sweep tests were conducted at 32 °C to examine the viscoelastic properties at administration conditions.

**Figure 7:**
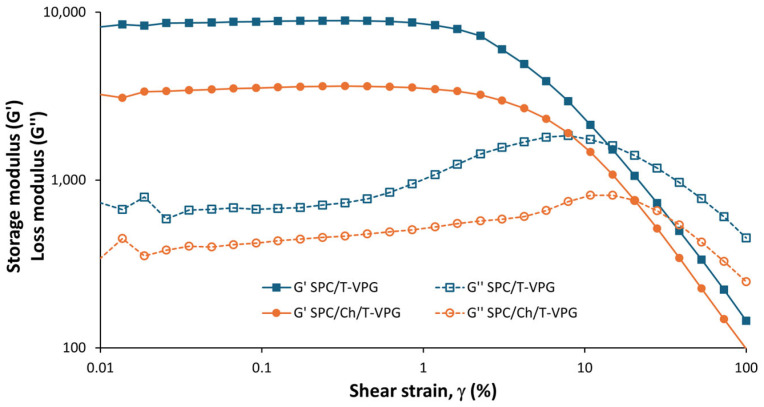
Amplitude sweep curves of T-VPGs at 32 °C.

As demonstrated in Figure 7, T-VPGs displayed a storage modulus (G′) greater than loss modulus (G″), indicating a viscoelastic solid-like structure of both VPGs. The linear viscoelastic region (characterized by a constant plateau where G′ and G″ remain unaffected by shear strain), was observed in the region from approximately 0.05 to 1%. However, the increase of shear strain over 1% resulted in a decrease of G″ and elevation of G′. When the shear strain surpassed roughly 10%, G″ also started to decrease, but this decline was milder compared to G′, and in that region, G′ became less than G″.

Comparison of these two T-VPGs showed higher values of G′ and G″ for SPC/T-VPG (Figure 7). This finding is contrary to expectations, as cholesterol, due to its heterocyclic ring structure, is incorporated within the hydrophobic tail region of phospholipid bilayers in liposomes, thereby increasing membrane rigidity and mechanical strength [52,53]. The possible reason for this outcome is the lower SPC ratio in SPC/Ch/T-VPG, since part of the SPC was replaced by cholesterol to maintain a constant total (phospho)lipid concentration (Table 1). While SPC is solely responsible for forming bilayers within the VPGs, decreasing its concentration also reduces the mechanical strength of SPC/Ch/T-VPG. A similar trend, where increasing SPC concentration led to higher overall viscosity of the VPG, was also reported by Tian et al. [54].

### 3.3. In Vitro Release of Thymol from T-VPGs

Among numerous parameters that determine the efficacy of topical dermatotherapy, an appropriate drug release pattern from the vehicle (formulation) is certainly one of the most important, as only the released and dissolved drug can exert a therapeutic effect. In addition, the mode of drug release over time is relevant for predicting the in vivo behavior of the drug, the onset of its action, and the required frequency of formulation application. Therefore, conducting in vitro release studies is fundamental during the development of T-VPGs. The in vitro release of thymol from T-VPGs was assessed under buffered conditions corresponding to the pH of the skin surface, using a dialysis tubing method (Section 2.5.).

**Figure 8:**
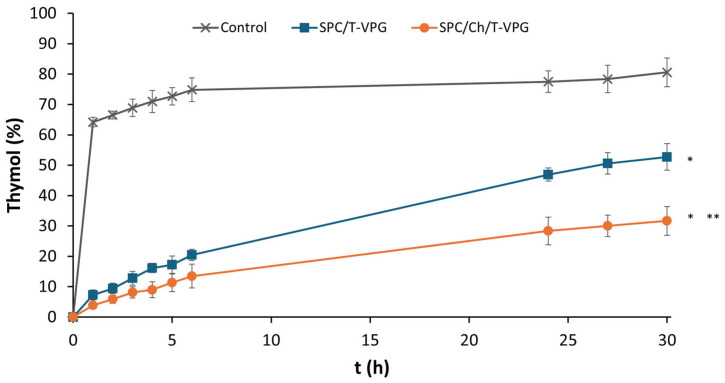
Cumulative release of thymol from different VPGs in phosphate buffer, pH 5.0. Values denote mean ± S.D. (n = 3). Free thymol (ethanol/water, 4/6, *v*/*v*), in same concentration of thymol as in T-VPGs, was used as a control. * Significantly different compared to control (ANOVA, *p* < 0.05). ** Significantly different compared to SPC/T-VPG (4–30 h; *t*-test, *p* < 0.05).

Both T-VPGs enabled prolonged release of thymol relative to the control (*p* < 0.05), which exhibited immediate thymol release (Figure 8). Comparison of the two T-VPGs demonstrated slower thymol release from the cholesterol-containing VPG due to increased rigidity of the phospholipid bilayers (Figure 2), significantly extending the release of the lipophilic active ingredient. Thus, even after 30 h, 32% of thymol was released from SPC/Ch/T-VPG, compared to 53% from SPC/T-VPG (*p* < 0.05). Such a release pattern is desirable for prolonging the local drug effect and reducing the frequency of formulation administration, which is especially important in the treatment of painful and inflamed areas of the skin.

In a previous study, in which a hydrophilic drug was entrapped into several VPGs differing in composition, controlled and prolonged release was also achieved. However, the amount of released drug was higher, particularly during the first 6 h of investigation, due to erosion of the VPG matrix and the release of a higher amount of hydrophilic drug located between neighboring vesicles, while bilayer fluidity was responsible for controlling the release of the liposomally entrapped drug [26].

Considering the structure of VPGs and the fact that they convert into VPG-liposomes upon exposure to an aqueous medium, it is assumed that the possible mechanisms involved in thymol release from T-VPGs include erosion of the VPG matrix and diffusion of thymol from the liposomal bilayers into the release medium.

The conducted studies also served to assess the kinetics of thymol release (Table 2). The optimal kinetic model was estimated based on the coefficients of determination (R^2^) for various models: Zero-order, First-order, Higuchi, and Korsmeyer–Peppas models [30,55]. The Higuchi model provided the best fit for both T-VPGs (R^2^ = 0.998), followed by the Korsmeyer–Peppas, with diffusion exponents of 0.61 and 0.62. These values indicate that thymol diffusion from T-VFGs into the release medium followed a non-Fickian diffusion mechanism. These results are in agreement with the study of El-Badry et al. [56], who evaluated the release of a lipophilic antifungal drug from liposomal gels.

### 3.4. Storage Stability Evaluation

Storage stability is an important factor to consider when developing drug (nano)formulations. The physical stability of T-VPGs was assessed by monitoring changes in the size and surface charge of T-VPG liposomes over 5 months storage at 4 °C.

**Figure 9:**
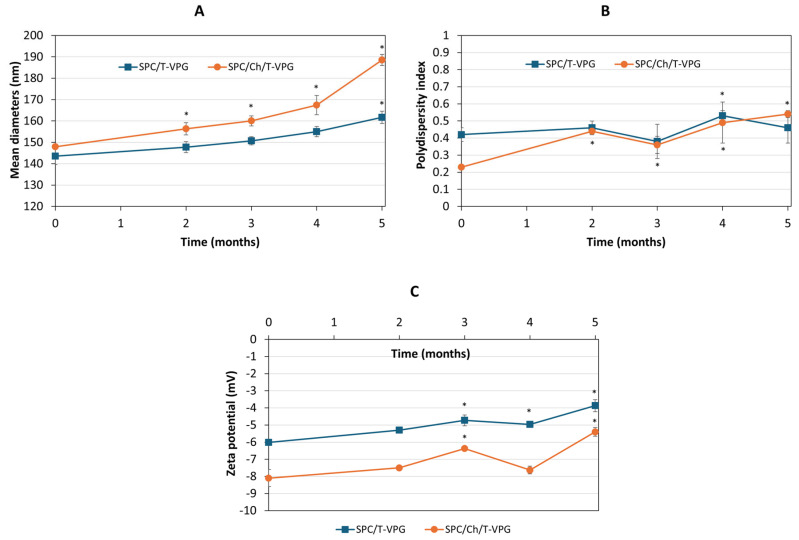
Storage stability profiles of T-VPGs: mean diameters (**A**), polydispersity indexes (**B**) and zeta potentials (**C**) of corresponding T-VPG liposomes. Values are mean ± S.D. (n = 3). * Significantly different compared to initial values (*t*-test, *p* < 0.05).

The results presented in Figure 9A,B demonstrate the stability of SPC/T-VPG liposomes for a period of 4 months, as their mean diameters and polydispersity indexes remained unchanged (*p* > 0.05). In contrast, SPC/Ch/T-VPG liposomes showed a significant increase in size after 2 months, which continued over the following 3 months. However, despite the significant increase over the period of 5 months, their mean diameters remained below 200 nm, which is an acceptable size for liposomes intended for dermal administration. Zeta potential values for both T-VPG liposomes shifted slightly over time toward a more neutral range (Figure 9C).

### 3.5. Ex Vivo Skin Penetration Studies

To better simulate dermal administration of T-VPGs and assess the capability of VPGs to localize thymol at the site of action, ex vivo skin penetration studies were performed. Additionally, the effect of bilayer fluidity of T-VPGs on thymol permeation into and through the skin was evaluated.

**Figure 10:**
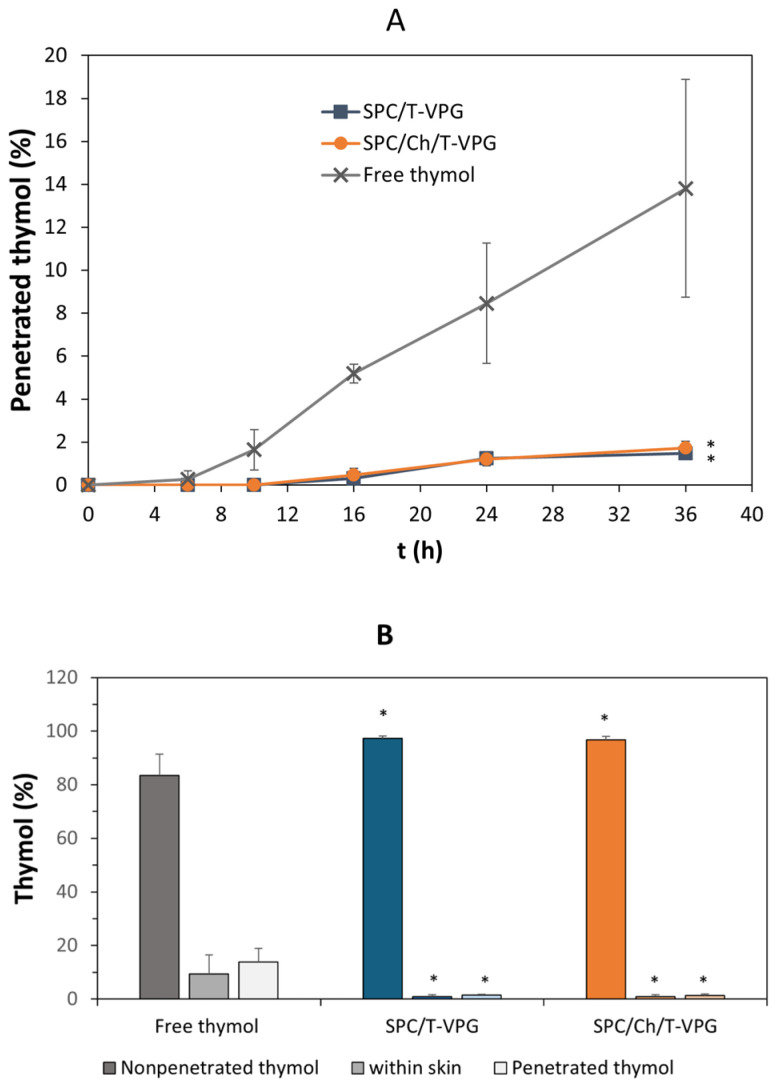
Cumulative amount of penetrated thymol through full thickness pig ear skin (**A**). Ex vivo accumulation/deposition/penetration of thymol on skin surface/within/through skin (**B**), corresponding to nonpenetrated thymol/thymol within skin/penetrated thymol. Values denote mean ± S.D. (n = 3). * Significantly different compared to free thymol (*p* < 0.05).

The results presented in Figure 10A clearly demonstrate the superiority of both T-VPGs in reducing the penetration rate of thymol through the skin compared to free thymol, thereby allowing its localization on the skin surface or within the uppermost skin layers (Figure 10B). No significant differences were observed in the amount of the penetrated, accumulated, or deposited thymol between the T-VPGs with differing bilayer fluidity (Figure 10). In contrast, the bilayer fluidity and viscosity of VPGs containing a hydrophilic drug influenced the localization site of the hydrophilic drug within the skin [26]. This inconsistency between the former [26] and the current study could be caused by significant differences in the viscosities and bilayer fluidities of the tested VPGs containing either a hydrophilic drug [26] or thymol. In the current study, both T-VPGs had comparable viscosities (Figure 6) and the difference in bilayer fluidity between the preparations was less pronounced than in the previous study [26]. In addition, the release of thymol was notably slower (Figure 8) compared to the release of the hydrophilic drug from VPGs of the same phospholipid composition [26], which significantly affected the penetration rate and deposition of thymol on the skin surface. Interestingly, although the amount of nonpenetrated thymol was also very high for the free thymol (Figure 10B), it was significantly lower compared to T-VPGs (*p* < 0.05).

### 3.6. In Vitro Antibacterial Assessment of T-VPG Liposomes

Thymol has been reported to exhibit a range of biological effects including antioxidant, antitumor, anti-inflammatory, immunomodulatory, antibacterial, antifungal, antiparasitic, and antiviral activities [1,2,3,4], making it a promising candidate for the treatment of various skin diseases. In this study, we focused on its antibacterial effect and investigated whether loading of thymol into VPG affected its antibacterial activity against the most common skin pathogen, *S. aureus*, as well as a resistant clinical isolate of *S. aureus*. The in vitro antibacterial potentials of T-VPG liposomes were compared with the activity of free thymol (ethanol/water solution, 4/6, *v*/*v*). All samples were tested at thymol concentrations ranging from 16 to 2048 µg/mL. The solvent used to dissolve thymol and reconstitute T-VPGs was also tested to exclude any spurious antibacterial effects caused by ethanol itself.

As shown in Table 3, MIC values for free thymol were determined to be 128 µg/mL for both tested strains. As an inoculum control, gentamicin was also tested, and the obtained MIC values correlated well with the literature data [57]. The antibacterial effect of T-VPG liposomes was observed only at the two highest tested thymol concentrations (2048 and 1024 µg/mL) for both strains. However, this effect was attributed to the antibacterial action of ethanol. At the next lower thymol concentration (512 µg/mL), T-VPG liposomes did not inhibit bacterial growth. Therefore, it was not possible to determine MIC values for T-VPG liposomes. We hypothesize that these difficulties in determining MIC values could be related to the observed precipitation of T-VPG liposomes in the presence of culture medium and bacteria, which likely made the liposomes less accessible for interaction with bacteria.

Surface charge of liposomes has been proven to affect the antibacterial activity of liposomes. Specifically, cationic liposomes have been shown to significantly enhance the antibacterial activity of liposomally-encapsulated azithromycin against *S. aureus* and MRSA strains due to strong electrostatic interaction of liposomes with the negatively surface-charged bacterial membrane [15,28,58]. In contrast, T-VPG liposomes in this study exhibited an almost neutral surface charge (Figure 3), limiting such interactions. Furthermore, the strong hydrophobicity of thymol and its high affinity for liposomal bilayers reduced its release into the bacterial environment. The aromatic ring and aliphatic side chains of thymol are thought to anchor it within the hydrophobic interior of the phospholipid bilayer, restricting thymol diffusion. Additionally, the small volume of culture medium used may not have supported sufficient thymol release to reach effective antibacterial concentrations. These factors, i.e., neutral surface charge (Figure 3), slow release of thymol (Figure 8), and low culture medium volume, probably contributed to the observed lack of antibacterial activity.

Reduced antibacterial activity of thymol liposomes relative to free thymol has also been reported by Heckler et al. [10]. In contrast, when VPG-liposomes containing ciprofloxacin hydrochloride were tested against the same bacterial strains [26], the activity of the liposomes was not hampered, and was equal to that of the free drug. Interestingly, there was no observed precipitation with those liposomes, despite being composed of the same (phospho)lipids. This was due to the positive surface charge of the ciprofloxacin hydrochloride overlapping the original neutral surface charge of the corresponding VPG-liposomes [26]. Therefore, future research should focus on developing positively charged liposomes with more fluidic bilayer properties to enhance liposome–bacteria interactions and increase thymol release rate.

### 3.7. Biocompatibility Evaluation of T-VPG Liposomes

Safety is one of the mandatory requirements for any drug (nano)formulation, regardless of its route of administration. Considering skin administration of T-VPGs, in vitro biocompatibility was assessed using keratinocyte monolayers. HaCaT cells were exposed to T-VPG liposomes containing thymol at concentrations ranging from 7.83 to 4000 µg/mL for 24 h, followed by determination of their metabolic activity using the MTT assay. As a control, free thymol was tested in the same concentration range, under the same conditions.

**Figure 11:**
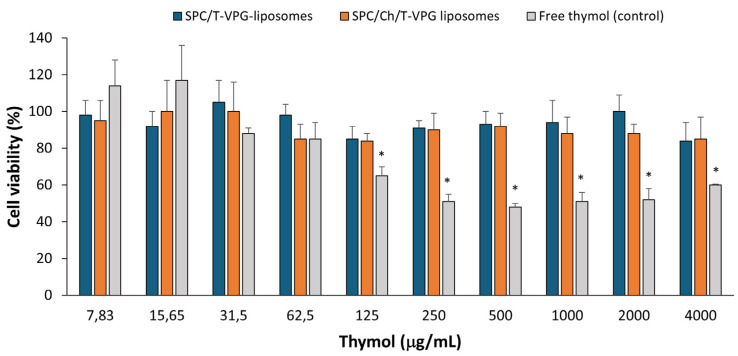
In vitro cytotoxicity of T-VPG liposomes and free thymol (control) towards HaCaT cells. Results are mean ± S.D. (n = 3). * Cell viability < 70%.

The results presented in Figure 11 demonstrate that both T-VPG liposomes were fully biocompatible with the cells in vitro. The tolerability of T-VPG liposomes by keratinocytes was confirmed even at an extremely high concentration of thymol (4 mg/mL) (cell viability ≥ 84%). A comparison of the different T-VPG liposomes proved that both nanoformulations were equally well-tolerated by the keratinocytes. Although negligible differences in the viability of HaCaT cells were observed after treatment with various T-VPG liposomes, they were not statistically significant (*p* > 0.05). The compatibility of the T-VPG liposomes with keratinocytes was somewhat expected due to their phospholipid composition and structural similarity to cellular membranes. Likewise, previous studies with liposomally-encapsulated antibiotics [26,28] also demonstrated the superiority of liposomes in reducing the cytotoxicity of the free drug.

In contrast to T-VPG liposomes, free thymol showed cytotoxic effects at concentrations from 125 µg/mL, with cell viability dropping below 70%. These cytotoxic effects correlate well with the antibacterial activity of thymol, as its MIC was determined to be 128 µg/mL (Table 3). It is assumed that thymol’s bactericidal activity occurs at concentrations exceeding this MIC, where its cytotoxic effects on keratinocytes become more pronounced. Therefore, encapsulation of thymol in VPG-liposomes significantly reduced its cytotoxicity (Figure 11). However, this encapsulation also interfered with the antibacterial efficacy of thymol (Table 3).

Interestingly, when tested at very low concentrations (7.83 and 15.65 µg/mL), free thymol demonstrated proliferative effects on keratinocytes (Figure 11). This is in accordance with the research by Folle et al. [8] who demonstrated in vitro biocompatibility of free thymol on keratinocytes at the concentrations of thymol 2–20 µg/mL.

### 3.8. In Vitro Healing Ability of T-VPGs

**Figure 12:**
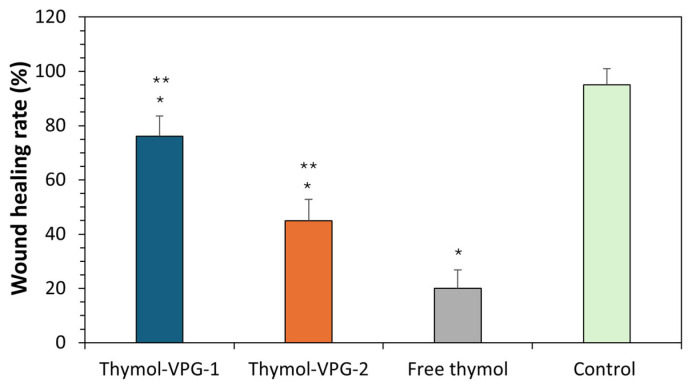
In vitro wound healing rate of T-VPG liposomes (determined 24 h after treatment). Concentration of thymol in all samples was 0.5 mg/mL. Control represented non-treated cells in culture medium. Values are mean ± S.D. (n = 3). * Significantly different compared to control (ANOVA, *p* < 0.05). ** Significantly different compared to free thymol (ANOVA, *p* < 0.05).

As a step forward toward the potential use of T-VPGs for the treatment of skin disorders or diseases involving a compromised epidermis, the effects of T-VPG liposomes on skin reepithelization were assessed. For this purpose, an in vitro method based on monitoring cells migration was applied to estimate the closure of the artificial gap in the keratinocytes’ monolayer [59]. The experiments were performed at thymol concentration that significantly exceeded its MIC value (500 µg/mL) and the results were expressed as the wound healing rate (Figure 12).

Encapsulation of thymol in VPG-liposomes significantly enhanced the closure of the damaged keratinocyte monolayer compared to free thymol (*p* < 0.05). Among the two tested T-VPG liposome formulations, SPC/T-VPG exhibited superior wound healing efficacy, probably due to the higher ratio of biocompatible phosphatidylcholine than in SPC/Ch/T-VPG liposomes. On the other hand, free thymol demonstrated a significant reduction in wound healing rate (Figure 12).

The concentration of thymol has shown to significantly influence wound closure. At low concentrations (3–10 µM), thymol has been reported to promote cell migration and enhance the healing process [4,8]. Conversely, when the cells were exposed to higher thymol concentrations (30 µM thymol), a meaningful reduction in the wound closure was observed [4]. Considering that the thymol concentration used in our study was substantially higher than those in previous investigations, the observed reduction in wound healing with free thymol is expected. These findings are consistent with the results reported by Moghtaderi et al. [60], where high concentrations of free thymol also negatively affected wound repair.

To better understand the therapeutic potential of T-VPGs, it would be appropriate to conduct in vivo studies to confirm their effects on the epithelialization process and overall pharmacological activity.

## 4. Conclusions

This research is the first to evaluate the potential of VPG as dermal vehicle for herbal-based medicines. Semisolid nanoformulations consisting of highly concentrated, small-sized liposomes capable of effectively encapsulating high amounts of thymol were prepared by a simple and robust method, utilizing a ‘green’ technology approach and enabling industrial-scale production. VPG proved to be a biocompatible vehicle with appropriate rheological properties, enabling localization of thymol in a large amount on the skin at the site of action. It remarkably reduced cytotoxic effects of thymol even when applied at very high concentrations, commonly required to achieve a therapeutic effect. Sustained and controlled release of thymol over a longer period would reduce the frequency of formulation administration, which is especially beneficial when treating painful skin conditions. Given the superior in vitro healing effect on a compromised epidermis and physical stability over a period of 4 months, SPC/T-VPG appears to be the favorable nanoformulation for dermal delivery of thymol. However, the attenuated antibacterial activity of thymol upon encapsulation remains a major challenge that requires further investigation.

## Figures and Tables

**Table 1:** Composition of different VPGs.

VPGs	SPC (g)	Cholesterol (g)	Thymol (g)	PG (g)	Water (g)
SPC-VPG	9.6	-	-	1	19.4
SPC/Ch-VPG *	8.6	1	-	1	19.4
SPC/T-VPG	9.6	-	0.5	1	18.9
SPC/Ch/T-VPG *	8.6	1	0.5	1	18.9

h, cholesterol; PG, propylene glycol; SPC, soybean lecithin with ≥ 94% phosphatidylcholine; T, thymol; VPG, vesicular phospholipid gel. * Procedure was performed at 50 °C. Total mass of each VPG was 30 g.

**Table 2:** Coefficients of determination (R^2^) for several kinetic models for thymol release from T-VPGs and corresponding Korsmeyer–Peppas diffusion exponents (n).

Kinetic Model	SPC/T-VPG	SPC/Ch/T-VPG
Zero-order	0.990	0.992
First-order	0.998	0.989
Higuchi model	0.998	0.998
Korsmeyer–Peppas	0.996 (n = 0.61)	0.995 (n = 0.62)

**Table 3:** In vitro antibacterial evaluation of T-VPG liposomes.

Sample	MIC (µg/mL)	
	*S. aureus* ATCC 6538	MRSA MFBF 10679
Free thymol	128	128
SPC/T-VPG liposomes	>512	>512
SPC/Ch/T-VPG liposomes	>512	>512
Gentamicin	0.25	0.50

## Data Availability

The original contributions presented in the study are included in the article; further inquiries can be directed to the corresponding author.

## References

[1] Gabbai-Armelin P.R., Sales L.S., Ferrisse T.M., De Oliveira A.B., De Oliveira J.R., Giro E.M.A., Brighenti F.L. (2022). A systematic review and meta-analysis of the effect of thymol as an anti-inflammatory and wound healing agent. Phytother. Res..

[2] Najafloo R., Behyari M., Imani R., Nour S. (2020). A mini-review of thymol incorporated materials: Applications in antibacterial wound dressing. J. Drug Deliv. Sci. Technol..

[3] Pivetta T.P., Simões S., Araújo M.M., Carvalho T., Arruda C., Marcato P.D. (2018). Development of nanoparticles from natural lipids for topical delivery of thymol: Investigation of its anti-inflammatory properties. Colloids Surf. B Biointerfaces.

[4] Lee K.P., Kim J.E., Park W.H., Hong H. (2016). Regulation of C6 glioma cell migration by thymol. Oncol. Lett..

[5] Pham Q.D., Björklund S., Engblom J., Topgaard D., Sparr E. (2016). Chemical penetration enhancers in stratum corneum—Relation between molecular effects and barrier function. J. Control. Release.

[6] (2024). Thymol. European Pharmacopeia 11.7.

[7] Garg A., Ahmad J., Hassan M.Z. (2021). Inclusion complex of thymol and hydroxypropyl-β-cyclodextrin (HP-β-CD) in polymeric hydrogel for topical application: Physicochemical characterization, molecular docking, and stability evaluation. J. Drug Deliv. Sci. Technol..

[8] Folle C., Marqués A.M., Díaz-Garrido N., Espina M., Sánchez-López E., Badia J., Baldoma L., Calpena A.C., García M.L. (2021). Thymol-loaded PLGA nanoparticles: An efficient approach for acne treatment. J. Nanobiotechnol..

[9] Deng L.L., Taxipalati M., Que F., Zhang H. (2016). Physical characterization and antioxidant activity of thymol solubilized Tween 80 micelles. Sci. Rep..

[10] Heckler C., Maders Silva C.M., Cacciatore F.A., Daroit D.J., da Silva Malheiros P. (2020). Thymol and carvacrol in nanoliposomes: Characterization and a comparison with free counterparts against planktonic and glass-adhered Salmonella. LWT.

[11] Gámez E., Elizondo-Castillo H., Tascon J., García-Salinas S., Navascues N., Mendoza G., Arruebo M., Irusta S. (2020). Antibacterial effect of thymol loaded SBA-15 nanorods incorporated in PCL electrospun fibers. Nanomaterials.

[12] Folle C., Sánchez-López E., Mallandrich M., Díaz-Garrido N., Suñer-Carbó J., Halbaut L., Carvajal-Vidal P., Marqués A.M., Espina M., Badia J. (2024). Semi-solid functionalized nanostructured lipid carriers loading thymol for skin disorders. Int. J. Pharm..

[13] Vanić Ž., Holæter A.M., Škalko-Basnet N. (2015). (Phospho)lipid-based nanosystems for skin administration. Curr. Pharm. Des..

[14] Van Tran V., Moon J.Y., Lee Y.C. (2019). Liposomes for delivery of antioxidants in cosmeceuticals: Challenges and development strategies. J. Control. Release.

[15] Rukavina Z., Jøraholmen M.W., Božić D., Frankol I., Golja Gašparović P., Škalko-Basnet N., Šegvić Klarić M., Vanić Ž. (2023). Azithromycin-loaded liposomal hydrogel: A step forward for enhanced treatment of MRSA-related skin infections. Acta Pharm..

[16] Čačić A., Amidžić Klarić D., Keser S., Radiković M., Rukavina Z., Jøraholmen M.W., Uzelac L., Kralj M., Škalko-Basnet N., Šegvić Klarić M. (2023). A novel approach for the treatment of aerobic vaginitis: Azithromycin liposomes-in-chitosan hydrogel. Pharmaceutics.

[17] Palac Z., Hurler J., Škalko-Basnet N., Filipović-Grčić J., Vanić Ž. (2015). Elastic liposomes-in-vehicle formulations destined for skin therapy: The synergy between type of liposomes and vehicle. Drug Dev. Ind. Pharm..

[18] Hemmingsen L.M., Giordani B., Pettersen A.K., Vitali B., Basnet P., Škalko-Basnet N. (2021). Liposomes-in-chitosan hydrogel boosts potential of chlorhexidine in biofilm eradication in vitro. Carbohydr. Polym..

[19] Brandl M., Drechsler M., Bachmann D., Bauer K.H. (1997). Morphology of semisolid aqueous phosphatidylcholine dispersions, a freeze fracture electron microscopy study. Chem. Phys. Lipids.

[20] Brandl M. (2007). Vesicular phospholipid gels: A technology platform. J. Liposome Res..

[21] Bender J., Michaelis W., Schubert R. (2002). Morphological and thermal properties of vesicular phospholipid gels studied by DSC, rheometry and electron microscopy. J. Therm. Anal. Calorim..

[22] Güthlein F., Burger A.M., Brandl M., Fiebig H.H., Schubert R., Unger C., Massing U. (2002). Pharmacokinetics and antitumor activity of vincristine entrapped in vesicular phospholipid gels. Anticancer Drugs.

[23] Kaiser N., Kimpfler A., Massing U., Burger A.M., Fiebig H.H., Brandl M., Schubert R. (2003). 5-Fluorouracil in vesicular phospholipid gels for anticancer treatment: Entrapment and release properties. Int. J. Pharm..

[24] Breitsamer M., Winter G. (2019). Vesicular phospholipid gels as drug delivery systems for small molecular weight drugs, peptides and proteins: State of the art review. Int. J. Pharm..

[25] Fang G., Wang Q., Yang X., Qian Y., Zhang G., Zhu Q., Tang B. (2021). Vesicular phospholipid gels as topical ocular delivery system for treatment of anterior uveitis. Colloid. Surf. A Physicochem. Eng. Asp..

[26] Keser S., Maravić-Vlahoviček G., Lovrić J., Vanić Ž. (2024). Vesicular phospholipid gels: A new strategy to improve topical antimicrobial dermatotherapy. Int. J. Pharm..

[27] (2024). Buffer solutions. European Pharmacopeia 11.7.

[28] Rukavina Z., Šegvić Klarić M., Filipović-Grčić J., Lovrić J., Vanić Ž. (2018). Azithromycin-loaded liposomes for enhanced topical treatment of methicillin-resistant *Staphylococcus aureus* (MRSA) infections. Int. J. Pharm..

[29] Vanić Ž., Rukavina Z., Manner S., Fallarero A., Uzelac L., Kralj M., Amidžić Klarić D., Bogdanov A., Raffai T., Virok D.P. (2019). Azithromycin-liposomes as a novel approach for localized therapy of cervicovaginal bacterial infections. Int. J. Nanomed..

[30] Bruschi M.L., Bruschi M.L. (2015). Mathematical models of drug. Strategies to Modify the Drug Release from Pharmaceutical Systems.

[31] Lu T., Ten Hagen T.L.M. (2020). A novel kinetic model to describe the ultra-fast triggered release of thermosensitive liposomal drug delivery systems. J. Control. Release.

[32] Weng J., Tong H.H.Y., Chow S.F. (2020). In vitro release study of the polymeric drug nanoparticles: Development and validation of a novel method. Pharmaceutics.

[33] NCCLS (2007). Performance Standards for Antimicrobial Susceptibility Testing.

[34] Klančnik A., Piskernik S., Jeršek B., Možina Smole S. (2010). Evaluation of diffusion and dilution methods to determine the antibacterial activity of plant extracts. J. Microbiol. Methods.

[35] Blažević F., Milekić T., Duvnjak Romić M., Juretić M., Pepić I., Filipović-Grčić J., Lovrić J., Hafner A. (2016). Nanoparticle-mediated interplay of chitosan and melatonin for improved wound epithelialisation. Carbohydr. Polym..

[36] Yagublu V., Karimova A., Hajibabazadeh J., Reissfelder C., Muradov M., Bellucci S., Allahverdiyev A. (2022). Overview of physicochemical properties of nanoparticles as drug carriers for targeted cancer therapy. J. Funct. Biomater..

[37] Raval N., Maheshwari R., Kalyane D., Youngren-Ortiz S.R., Chougule M.B., Tekade R.K., Tekade R.K. (2019). Chapter 10—Importance of physicochemical characterization of nanoparticles in pharmaceutical product development. Advances in Pharmaceutical Product Development and Research Basic Fundamentals of Drug Delivery.

[38] Vanić Ž., Hurler J., Ferderber K., Golja Gašparović P., Škalko-Basnet N., Filipović-Grčić J. (2014). Novel vaginal drug delivery system: Deformable propylene glycol liposomes-in-hydrogel. J. Liposome Res..

[39] Ibaraki H., Kanazawa T., Oogia C., Takashima Y., Seta Y. (2019). Effects of surface charge and flexibility of liposomes on dermal drug delivery. J. Drug Deliv. Sci. Technol..

[40] Baldassarre F., Schiavi D., Ciarroni S., Tagliavento V., De Stradis A., Vergaro V., Suranna G.P., Balestra G.M., Ciccarella G. (2023). Thymol-nanoparticles as effective biocides against the quarantine pathogen *Xylella fastidiosa*. Nanomaterials.

[41] Engel J.B., Heckler C., Tondo E.C., Daroit D.J., da Silva Malheiros P. (2017). Antimicrobial activity of free and liposome-encapsulated thymol and carvacrol against *Salmonella* and *Staphylococcus aureus* adhered to stainless steel. Int. J. Food Microbiol..

[42] Miranda-Cadena K., Dias M., Costa-Barbosa A., Collins T., Marcos-Arias C., Eraso E., Pais C., Quindós G., Sampaio P. (2021). Development and characterization of monoolein-based liposomes of carvacrol, cinnamaldehyde, citral, or thymol with anti-candida activities. Antimicrob. Agents Chemother..

[43] Proksch E. (2018). pH in nature, humans and skin. J. Dermatol..

[44] Brooks S.G., Mahmoud R.H., Lin R.R., Fluhr J.W., Yosipovitch G. (2025). The skin acid mantle: An update on skin pH. J. Investig. Dermatol..

[45] Sim P., Strudwick X.L., Song Y., Cowin A.J., Garg S. (2022). Influence of Acidic pH on wound healing in vivo: A novel perspective for wound treatment. Int. J. Mol. Sci..

[46] Lukić M., Pantelić I., Savić S.D. (2021). Towards optimal pH of the skin and topical formulations: From the current state of the art to tailored products. Cosmetics.

[47] Grit M., Crommelin D.J. (1993). Chemical stability of liposomes: Implications for their physical stability. Chem. Phys. Lipids.

[48] Qwist P.K., Sander C., Okkels F., Jessen V., Baldursdottir S., Rantanen J. (2019). On-line rheological characterization of semi-solid formulations. Eur. J. Pharm. Sci..

[49] Alves M.P., Raffin R.P., Fagan S.B., Beck R., Guterres S., Pohlmann A. (2011). Rheological behavior of semisolid formulations containing nanostructured systems. Nanocosmetics and Nanomedicines.

[50] Simões A., Miranda M., Cardoso C., Vitorino F.V.A. (2020). Rheology by design: A regulatory tutorial for analytical method validation. Pharmaceutics.

[51] Barnes H.A. (2000). A Handbook of Elementary Rheology.

[52] Najafinobar N., Mellander L.J., Kurczy M.E., Dunevall J., Angerer T.B., Fletcher J.S., Cans A.S. (2016). Cholesterol alters the dynamics of release in protein independent cell models for exocytosis. Sci. Rep..

[53] Kotla N.G., Chandrasekar B., Rooney P., Sivaraman G., Larrañaga A., Krishna K.V., Pandit A., Rochev Y. (2017). Biomimetic lipid-based nanosystems for enhanced dermal delivery of drugs and bioactive agents. ACS Biomater. Sci Eng..

[54] Tian W., Schulze S., Brandl M., Winter G. (2010). Vesicular phospholipid gel-based depot formulations for pharmaceutical proteins: Development and in vitro evaluation. J. Control. Release.

[55] Duvnjak Romić M., Šegvić Klarić M., Lovrić J., Pepić I., Cetina-Čižmek B., Filipović-Grčić J., Hafner A. (2016). Melatonin-loaded chitosan/Pluronic^®^ F127 microspheres as in situ forming hydrogel: An innovative antimicrobial wound dressing. Eur. J. Pharm. Biopharm..

[56] El-Badry M., Fetih G., Shakeel F. (2014). Comparative topical delivery of antifungal drug croconazole using liposome and micro-emulsion-based gel formulations. Drug Deliv..

[57] European Society of Clinical Microbiology and Infectious Diseases (EUCAST) (2003). Determination of minimum inhibitory concentrations (MICs) of antibacterial agents by broth dilution. Clin. Microbiol. Infect..

[58] Bogdanov A., Janovák L., Vraneš J., Meštrović T., Ljubin-Sternak S., Cseh Z., Endrész V., Burián K., Vanić Ž., Virok D.P. (2022). Liposomal encapsulation increases the efficacy of azithromycin against *Chlamydia trachomatis*. Pharmaceutics.

[59] Moghtaderi M., Bazzazan S., Sorourian G., Sorourian M., Akhavanzanjani Y., Noorbazargan H., Ren Q. (2023). Encapsulation of thymol in gelatin methacryloyl (gelma)-based nanoniosome enables enhanced antibiofilm activity and wound healing. Pharmaceutics.

[60] Balko S., Kerr E., Buchel E., Logsetty S., Raouf A. (2023). A robust and standardized approach to quantify wound closure using the scratch assay. Methods Protoc..

